# NCF2 facilitates M2 macrophage polarization in glioblastoma through activation of the notch1–osteopontin axis

**DOI:** 10.3389/fimmu.2026.1743950

**Published:** 2026-02-12

**Authors:** Yinsong Zhang, Na Li, Hongbo Cheng, Yi Xiang, Guozhu Sun

**Affiliations:** 1 Department of Neurosurgery, The Second Hospital of Hebei Medical University, Shijiazhuang, Hebei, China; 2 Department of Neurosurgery, Hengshui People’s Hospital, Hengshui, Hebei, China; 3 Department of Medical Oncology, Hengshui People’s Hospital, Hengshui, Hebei, China

**Keywords:** GBM, macrophage polarization, ncf2, NOTCH1, OPN, tumor microenvironment

## Abstract

**Background:**

Glioblastoma (GBM), the most aggressive form of primary malignant brain neoplasm, is characterized by extensive invasiveness, high recurrence rates, and poor clinical outcomes. Tumor-associated macrophages, particularly those exhibiting an alternatively activated (M2) phenotype, play a critical role in promoting progression within the GBM environment. However, the molecular mechanisms underlying macrophage polarization in GBM remain insufficiently defined.

**Methods:**

The expression of neutrophil cytosolic factor 2 (NCF2) was evaluated in GBM tissue specimens and cell lines using immunohistochemistry, western blot analysis, and reverse transcription polymerase chain reaction. Associations between NCF2, the M2 macrophage marker CD163, and osteopontin (OPN) were analyzed in 18 GBM specimens. Functional studies were performed by co-culturing human monocyte-derived macrophages with GBM cells that either overexpressed or exhibited silenced NCF2 expression. The roles of Notch1 signaling and OPN in NCF2-mediated macrophage polarization were evaluated using pharmacological inhibitors and neutralizing antibodies.

**Results:**

NCF2 expression was significantly elevated in GBM tissues and cell lines and demonstrated a positive correlation with CD163+ M2 macrophage infiltration (r = 0.765, *p* < 0.001). *In vitro* assays indicated that NCF2 promoted polarization toward the M2 phenotype, as indicated by upregulation of CD163 and CCL18, without affecting proinflammatory markers such as TNF-α or CD86. NCF2 levels correlated positively with OPN expression (r = 0.745, *p* < 0.001), and OPN was identified as a downstream target regulated through the Notch1 pathway. Activation of Notch1 restored OPN expression in *NCF2*-silenced cells, whereas its inhibition reduced OPN expression in NCF2-overexpressing cells. Neutralization of OPN reversed the M2-polarizing effect induced by NCF2.

**Conclusion:**

NCF2 contributes to the establishment of an immunosuppressive tumor microenvironment in GBM by promoting macrophage polarization via the Notch1–OPN pathway. These findings highlight NCF2 as a potential molecular target for therapeutic intervention in GBM.

## 1 Introduction

Glioma represents the most common malignant neoplasm of the central nervous system, accounting for approximately 80% of all primary malignant brain tumors. The global incidence of glioma is estimated at 6 cases per 100,000 individuals annually. GBM is the most aggressive and prevalent type of glioma, characterized by a high rate of recurrence and a markedly invasive phenotype. Despite advancements in neurosurgical techniques, radiotherapy, and chemotherapy, clinical outcomes for individuals diagnosed with GBM remain poor. The median overall survival ranges from 12 to 15 months, and the 5-year survival rate remains below 10% (1). Consequently, investigating the molecular pathways underlying GBM pathogenesis is essential to identify new therapeutic targets and improve clinical outcomes (2).

Neutrophil cytosolic factor 2 (NCF2), also referred to as NOXA2 or p67phox, is a cytoplasmic non-glycosylated regulatory subunit of the nicotinamide adenine dinucleotide phosphate (NADPH) oxidase complex. NCF2 plays an important role in the generation of reactive oxygen species (ROS) and has been implicated in tumor initiation and progression. Studies have demonstrated that NCF2 predominantly exerts pro-tumorigenic effects across various malignancies (3–6). However, most studies have focused on the correlation between NCF2 expression levels and clinical prognosis, while there has been limited exploration of the underlying molecular mechanisms, particularly in the context of GBM.

Recent investigations have reported a positive association between elevated NCF2 expression and increased infiltration of alternatively activated (M2) tumor-associated macrophages (TAMS), which is in turn associated with poorer clinical prognosis (3). TAMs, especially those exhibiting the M2 phenotype, are recognized as critical mediators of glioma proliferation, invasion, and immune suppression (7, 8). Nonetheless, it remains unclear whether NCF2 directly regulates macrophage polarization in the GBM microenvironment, and the signaling pathways involved in this process remain unidentified.

The present study aims to study the functional role of NCF2 in GBM progression, with a particular focus on its interaction with TAMs. In particular, this work seeks to determine whether NCF2 regulates M2 macrophage polarization and to delineate the involvement of the Notch1/osteopontin (OPN) signaling axis in this regulatory mechanism. These findings may provide novel insights into the immunoregulatory role of NCF2 in GBM and contribute to the identification of new molecular targets for therapeutic intervention.

## 2 Materials and methods

### 2.1 Clinical samples

GBM tissue samples (n = 18) were obtained from patients undergoing neurosurgical resection at the Second Hospital of Hebei Medical University. None of the patients had received chemotherapy or radiotherapy prior to surgery. As a control group, histologically normal brain tissues (n = 17) were collected from patients undergoing craniotomy for traumatic brain injury. All tissue specimens were fixed in 4% paraformaldehyde, paraffin-embedded, and processed for immunohistochemistry RNA extraction, and protein analysis. This study was approved by the institutional Ethics Committee, and written informed consent was obtained from all participants.

### 2.2 Immunohistochemical analysis

Formalin-fixed, paraffin-embedded tissue sections (4 μm thick) were deparaffinized in xylene and rehydrated. Antigen retrieval was performed using citrate buffer (pH 6.0). Endogenous peroxidase activity was blocked using 3% hydrogen peroxide for 15 minutes. Sections were incubated overnight at 4°C with primary antibodies specific for NCF2(1:500,ab109366), CD163(1:500,ab316218), and OPN(1:100,AF0227). After washing, horseradish peroxidase (HRP)-conjugated secondary antibodies were applied. Immunoreactivity was visualized using 3,3′-diaminobenzidine and counterstained with hematoxylin. The staining intensity is categorized as follows: no staining = 0 points, weak staining = 1 point, moderate staining = 2 points, and strong staining = 3 points.

The proportion of positive cells is classified as: <1% = 0 points, 1%–33% = 1 point, 33%–67% = 2 points, and >67% = 3 points. The final score is calculated by multiplying the scores for staining intensity and the proportion of positive cells. Based on the final score, expression is typically graded as: 0 points = negative (−), 1–2 points = weakly positive (+), 3–4 points = moderately positive (++), and 5–9 points = strongly positive (+++).The positive expression rate of NCF2 was calculated as the average of the ratios of positive cells to total cells in five randomly selected fields of view at 20× magnification. All sections were evaluated independently and blindly by two experienced pathologists.

### 2.3 Cell culture

Human GBM cell lines (U251, U87, LN229, and T98G) and normal human astrocytes (HA) were procured from the Cell Bank of the Chinese Academy of Sciences (Shanghai, China). Cells were cultured in Dulbecco’s Modified Eagle Medium (Gibco, USA) supplemented with 10% fetal bovine serum (FBS), 100 U/mL penicillin, and 100 µg/mL streptomycin. All cells were maintained at 37 °C in a humidified incubator with 5% CO_2_.

### 2.4 Plasmid construction, siRNA, and transfection

A mammalian expression plasmid encoding the full-length open reading frame (ORF) of human *NCF2* (NM_000433) tagged with Myc-DDK (FLAG) was obtained from OriGene (catalog no. RC200704, Rockville, MD, USA). For RNA interference, pre-designed Silencer Select small interfering RNAs (siRNAs) targeting human *NCF2*, along with a non-targeting negative control siRNA (#4390847), were purchased from Thermo Fisher Scientific (Waltham, MA, USA). Cells were seeded in six-well plates and transfected at 60%–70% confluence. For plasmid overexpression, 2–3 µg of DNA per well was transfected using Lipofectamine 3000 (Invitrogen, Carlsbad, CA, USA), following the manufacturer’s instructions. For gene silencing, siRNAs were transfected using Lipofectamine RNAiMAX (Invitrogen) at a final concentration of 20 nM. Cells were harvested 24–48 hours post-transfection for plasmid-based experiments and 48–72 hours post-transfection for siRNA experiments. Samples were subsequently used for quantitative reverse transcription PCR (qRT-PCR), western blotting, and functional assays.

### 2.5 Western blot analysis

Total protein was extracted using RIPA lysis buffer containing protease and phosphatase inhibitors (Beyotime, Shanghai, China). Protein concentrations were quantified using the BCA assay (Thermo fisher Scientific, USA). Equal amounts of protein (20 to 30 µg) were separated by sodium dodecyl sulfate–polyacrylamide gel electrophoresis and subsequently transferred to polyvinylidene difluoride membranes (Millipore, USA). After blocking with 5% nonfat milk, for 1 hour at room temperature, membranes were incubated overnight at 4°C with primary antibodies targeting NCF2(1:5000,ab109366), OPN(1:1000,AF0227), Notch1(1:1000,ab52627), and β-actin (1:10000,AF7018). Following incubation with HRP-conjugated secondary antibodies, protein bands were visualized using an enhanced chemiluminescence detection system.

### 2.6 Quantitative real-time PCR

Total RNA was extracted from GBM cells using TRIzol reagent (Invitrogen, USA). Complementary DNA(cDNA) was synthesized from 1 µg of total RNA using the PrimeScript RT Reagent Kit (GenStar,China). Quantitative PCR track was performed using SYBR Green Master Mix (GenStar) on a StepOnePlusReal-Time PCR System. Relative gene expression was calculated using the 2^-ΔΔCt method,with β-actin serving as the internal reference control. Each experimental group included atleast three independent biological replicates, and for each independent cDNA sample, three technicalreplicates were performed during quantitative polymerase chain reaction (qPCR). The cycle threshold (Ct) values were averaged for subsequent calculations. Primer sequences are provided in **Supplementary Table S1**.

### 2.7 Isolation and differentiation of human monocytes/macrophages

Peripheral blood mononuclear cells (PBMCs) were isolated from the peripheral blood of healthy donors by density gradient centrifugation using Ficoll-Paque PLUS (GE Healthcare, USA). Monocytes were isolated through adherence to plastic surfaces and culture in RPMI-1640 medium supplemented with 10% FBS and 50 ng/mL M-CSF (PeproTech, USA) for 7 days to facilitate differentiation into macrophages (9).

### 2.8 Conditioned medium and macrophage co-culture

GBM cells (control, NCF2-overexpressing, or NCF2-knockdown) were cultured for 48 hours, after which supernatants were collected and used as conditioned medium (CM). Differentiated macrophages were exposed to the CM medium for 24 hours. To assess the functional role of OPN, a neutralizing anti-OPN antibody (R&D Systems, USA) or an isotype-matched control antibody was added to the CM during co-culture.

### 2.9 Immunofluorescence analysis

Macrophages were seeded onto glass coverslips, fixed in 4% paraformaldehyde for 15 minutes, permeabilized with 0.1% Triton X-100, and blocked with 3% BSA. Cells were incubated with primary antibodies against CD163(1:50,ab316218) and CD86(1:100,ab239075), followed by Alexa Fluor-conjugated secondary antibodies. Nuclei were counterstained with 4′,6-diamidino-2-phenylindole. Fluorescence images were obtained using a confocal laser scanning microscope (Leica Microsystems, Germany).

### 2.10 Notch1 pathway modulation

To investigate the involvement of the Notch1 signaling pathway, NCF2-knockdown or NCF2-overexpressing GBM cells were treated with either a Notch1 agonist (recombinant human Jagged1, 2 µg/mL; AF1277;R&D Systems) or a Notch1 inhibitor (DAPT, 10 µM; 2634; R&D Systems) for 48 h. Following treatment, the protein and mRNA expression levels of Notch1 and OPN were analyzed by western blot and qRT-PCR.

### 2.11 Statistical analysis

Data are presented as mean ± standard deviation (SD). Comparisons of categorical variables were conducted utilizing the chi-squared test. Spearman’s rank correlation coefficient was used to analyze the relationship between NCF2 expression and levels of CD163 or OPN. A two-tailed *p*-value of <0.05 was considered statistically significant. All statistical analyses were performed using SPSS software version 23.0 (IBM Corp., Armonk, NY, USA).

### 2.12 GEPIA database expression analysis

Gene expression correlations between NCF2 and OPN (encoded by SPP1), as well as between NCF2 and CD163, were analyzed using the GEPIA database. Pearson correlation analysis was performed on log2(TPM + 1)-transformed mRNA expression data from The Cancer Genome Atlas (TCGA) tumor samples. Statistical significance was set at p < 0.05. The analysis produced a scatter plot with the calculated Pearson’s R coefficient, p-value, and regression line to evaluate the strength and direction of the correlation.

## 3 Results

### 3.1 NCF2 is highly expressed in GBM

**Figure 1:**
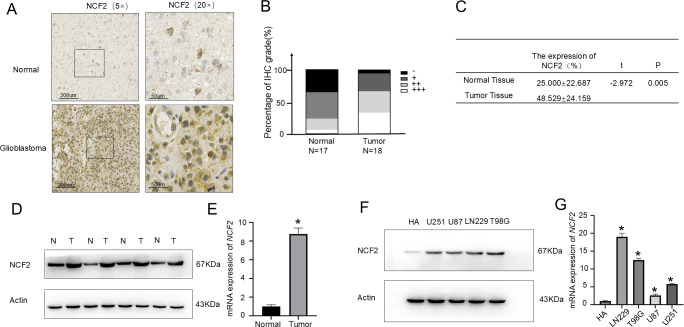
NCF2 expression is elevated in GBM compared with normal brain tissues. **(A)** Representative immunohistochemistry (IHC) staining showing NCF2 expression in normal brain tissues (upper panel) and GBM tissues (lower panel). **(B)** Proportional distribution of NCF2 expression levels across all examined tissue specimens, presented as a bar chart. **(C)** Comparative analysis of NCF2 IHC staining intensity between GBM and normal brain tissue (t=-2.972, *P* = 0.005). **(D)** Western blot validation of NCF2 protein expression in GBM tissues versus normal brain tissues. **(E)** Quantitative real-time PCR (qRT-PCR) validation of *NCF2* mRNA expression in GBM tissues versus normal brain tissues. qPCR was performed in triplicate for each sample. **(F)** Western blot analysis of NCF2 protein levels in GBM cell lines compared with normal human astrocytes. **(G)** qRT-PCR validation of *NCF2* mRNA expression in GBM cell lines compared with normal human astrocytes. qPCR was performed in triplicate for each sample.(“*”represents P<0.05).

IHC analysis was conducted on 18 GBM tissue samples and 17 histologically normal brain tissues. The results demonstrated significantly elevated NCF2 expression in GBM tissues compared with normal controls (**Figure 1A**). Semi-quantitative scoring indicated a higher proportion of cases with strong NCF2 immunoreactivity in GBM than in normal brain tissues (**Figure 1B**). This difference was statistically significant (t=-2.972; P = 0.005 **Figure 1C**). Western blot analysis further confirmed increased NCF2 protein levels in GBM tissues (n = 4) relative to normal brain tissues (n = 4) (**Figure 1D**). Similarly, qRT-PCR analysis indicated that *NCF2* mRNA expression was increased in GBM tissues compared to normal controls (Change multiplier = 8.17, P = 0.000) (**Figure 1E**). At the cellular level, western blotting indicated that NCF2 protein expression was markedly higher in GBM cell lines (U251, U87, LN229, and T98G) compared with normal human astrocytes (HA) (**Figure 1F**). Consistently, *NCF2* mRNA levels were also significantly increased in GBM cell lines (LN229, T98G, U87, U251) relative to HA cells (Change multiplier = 19.663,12.236, 2.558,5.818, P = 0.000) (**Figure 1G**).

### 3.2 NCF2 expression correlates with local infiltration of M2 macrophages

**Figure 2:**
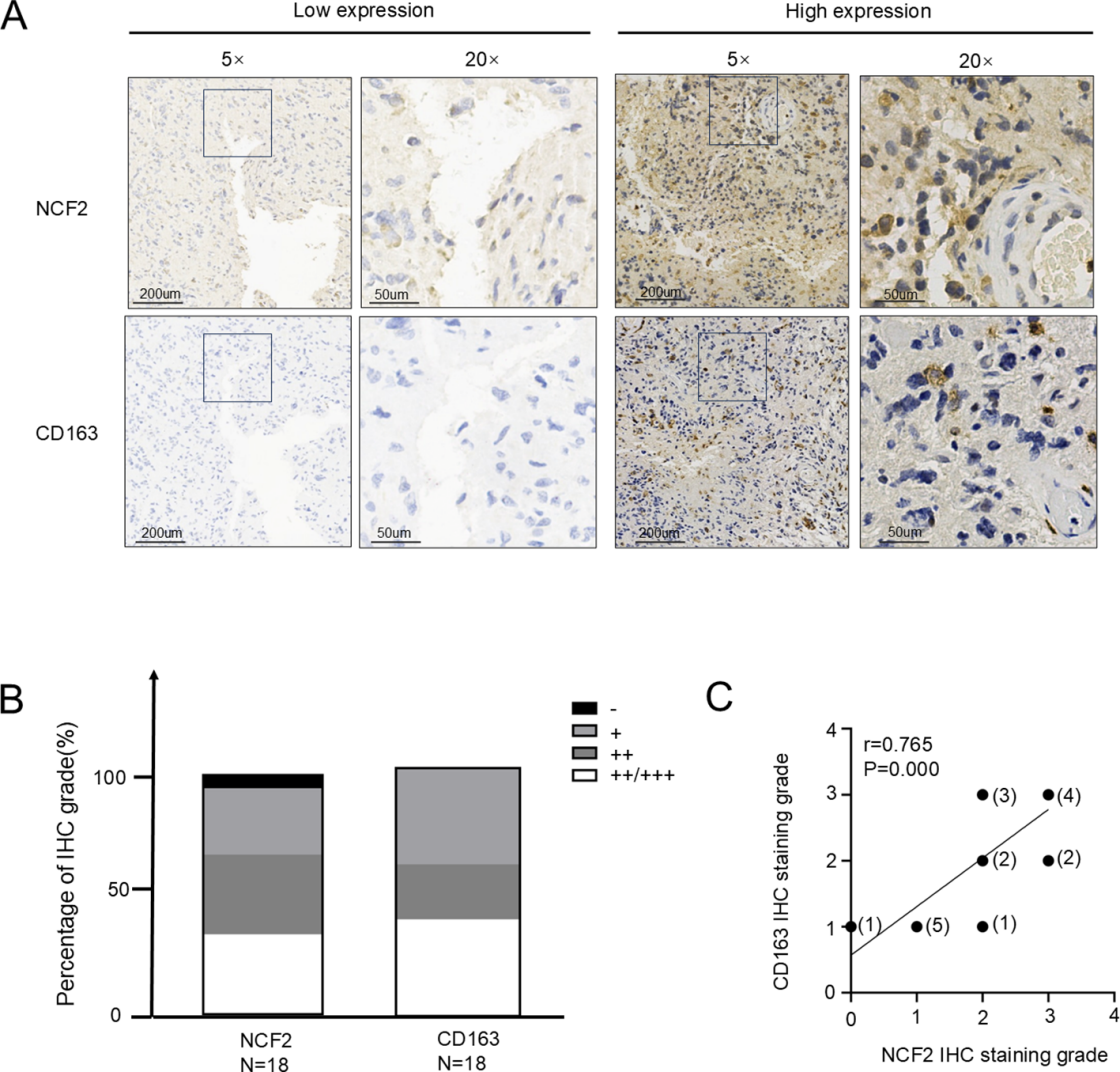
NCF2 expression in GBM tissues is significantly correlated with the infiltration of CD163^+^ macrophages. **(A)** Representative immunohistochemistry (IHC) staining showing NCF2 expression (upper panel) and CD163 expression (lower panel) in GBM tissues. **(B)** Distribution of NCF2 and CD163 expression levels across all GBM tissue specimens, presented as bar charts. **(C)** Spearman’s correlation analysis of IHC staining scores between NCF2 and CD163 in GBM tissues (The number above each data point represents the specific number of samples corresponding to that expression level) (r = 0.765, P = 0.000).

To examine the relationship between NCF2 expression and macrophage polarization, IHC staining for NCF2 and the M2 macrophage marker CD163 was performed on 18 GBM specimens. Tumors exhibiting low NCF2 expression showed markedly reduced infiltration of CD163^+^ macrophages, whereas those with high NCF2 expression displayed significantly increased CD163^+^ cell infiltration (**Figure 2A**). Immunohistochemical analysis of NCF2 and CD163 expression in consecutive tissue sections revealed a high degree of concordance in their expression levels. (**Figure 2B**). Spearman’s correlation analysis demonstrated a strong positive association between NCF2 and CD163 expression levels (r = 0.765, *p* < 0.001; **Figure 2C**). GEPIA is an interactive analysis platform based on gene expression level values. On this platform, a significant correlation (P = 0.00,R = 0.72) was found between the mRNA expression levels of *NCF2* and *CD163* (**Supplementary Figure S3**).

### 3.3 NCF2 promotes macrophage polarization toward an M2 phenotype

**Figure 3:**
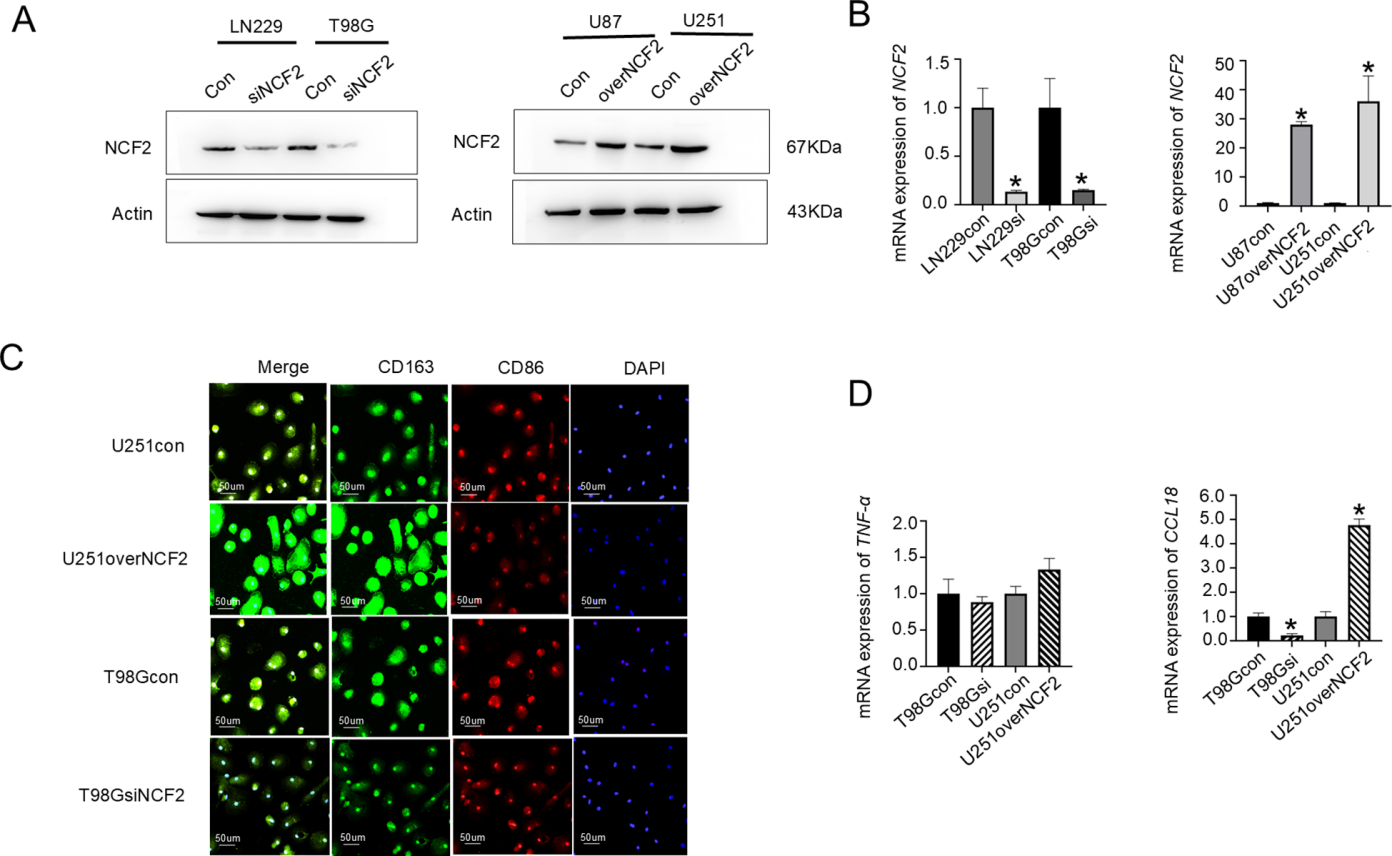
NCF2 promotes macrophage polarization toward the M2 phenotype. **(A)** Western blotvalidation of NCF2 protein expression in LN229 and T98G cells with NCF2 knockdown, and in U87 andU251 cells with NCF2 overexpression. **(B)** qRT-PCR validation of *NCF2*mRNA expression in LN229 and T98G cells with NCF2 knockdown, and in U87 and U251 cells with NCF2overexpression. qPCR was performed in triplicate for each sample. **(C)** Representativeimmunofluorescence staining of macrophages after 24 h co-culture with conditioned media from U251 cells overexpressing NCF2, T98G cells with NCF2 knockdown, or control cells, showing the expression of CD163 (M2 marker) and CD86 (M1 marker). Macrophages were differentiated from peripheral blood mononuclear cells (PBMCs) isolated from healthy donors (illustrated in **Supplementary Figure S1**). **(D)** qRT-PCR analysis of *TNF-α* (M1 marker) and *CCL18* (M2 marker) mRNA expression in macrophages after 24 h co-culture with conditioned media from U251 cells overexpressing NCF2, T98G cells with NCF2 knockdown, or control cells. qPCR was performed in triplicate for each sample. (“*”represents P<0.05).

Stable GBM cell lines with NCF2 knockdown (LN229 and T98G) and overexpression (U87 and U251) were established and validated by both western blot and qRT-PCR(LN229, T98G, U87, U251) (Change multiplier=0.103,P=0.016; 0.108,P = 0.009; 28,P = 0.000; 36,P = 0.020) (**Figures 3A, B**). PBMCs from healthy donors were differentiated into macrophages and co-cultured with conditioned media derived from the genetically modified GBM cells. Immunofluorescence staining revealed that macrophages exposed to supernatants from NCF2-overexpressing U251 cells exhibited markedly increased CD163 expression, while those exposed to supernatants from NCF2-knockdown T98G cells displayed reduced CD163 expression. CD86 expression remained largely unchanged across groups (**Figure 3C**). qRT-PCR analysis indicated no significant change in *TNF-α* mRNA levels; however, *CCL18* expression was significantly upregulated in macrophages exposed to *NCF2*-overexpressing U251 supernatants and downregulated in macrophages exposed to *NCF2*-knockdown T98G supernatants(T98Gsi,U251over) (TNF-α:Change multiplier=0.883,P=0.415; 1.333, P = 0.051; CCL18:Change multiplier=0.223,P=0.004; 4.767,P = 0.000) (**Figure 3D**). These findings indicate that NCF2 facilitates macrophage polarization toward an M2-like immunosuppressive phenotype.

### 3.4 NCF2 expression is positively correlated with OPN levels in GBM

**Figure 4:**
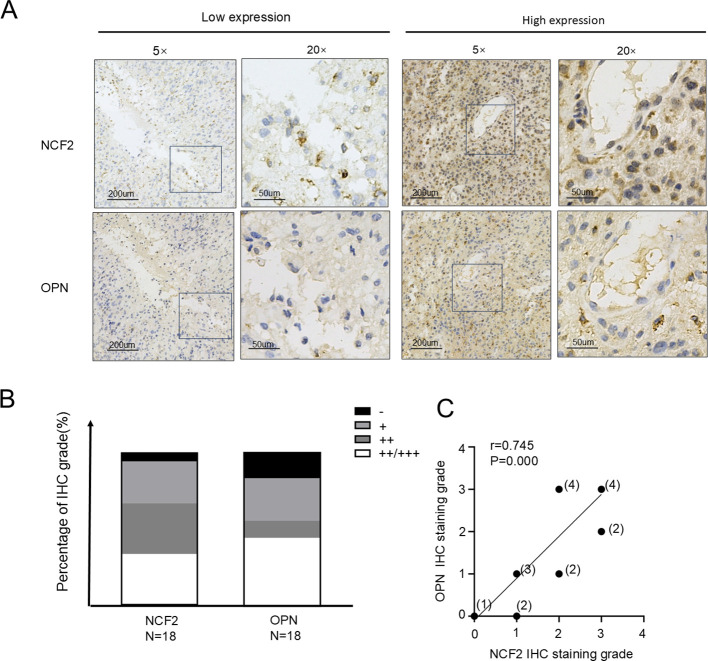
NCF2 expression in GBM tissues is significantly correlated with OPN expression. **(A)** Representative immunohistochemistry (IHC) staining showing NCF2 expression (upper panel) and OPN expression (lower panel) in GBM tissues. **(B)** Distribution of NCF2 and OPN expression levels across all GBM tissue specimens, presented as bar charts. **(C)** Spearman’s correlation analysis of IHC staining scores between NCF2 and OPN in GBM tissues (The number above each data point represents the specific number of samples corresponding to that expression level) (r = 0.745, P = 0.000). OPN (osteopontin) is an macrophage polarization–related factor, suggesting a potential mechanistic link between NCF2 expression and macrophage phenotype regulation.

To further explore the molecular association between NCF2 and OPN, IHC analysis was performed on the same set of 18 GBM tissue specimens. Tumors with low NCF2 expression exhibited reduced OPN levels, while those with high NCF2 expression demonstrated increased OPN expression (**Figure 4A**). The distribution patterns of NCF2 and OPN were similar (**Figure 4B**). Spearman’s correlation analysis confirmed a significant positive association between NCF2 and OPN expression (r = 0.745, *p* < 0.001; **Figure 4C**). GEPIA is an interactive analysis platform based on gene expression level values. On this platform, a significant correlation (P = 4.4e-16,R=0.58) was found between the mRNA expression levels of *NCF2* and *OPN* (**Supplementary Figure S2**).

### 3.5 NCF2 regulates OPN expression through Notch1 signaling

**Figure 5:**
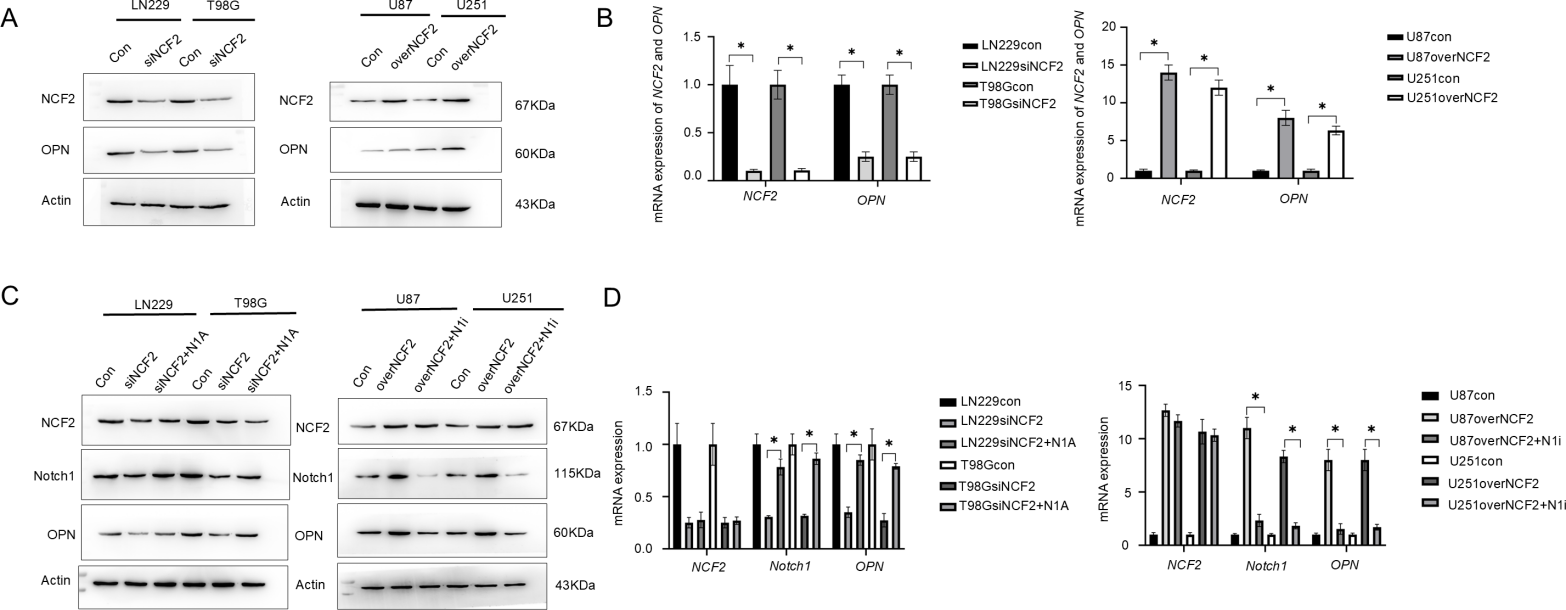
NCF2 regulates OPN expression via Notch1 signaling in GBM. **(A)** Western blot analysis showing NCF2 and OPN protein expression in LN229 and T98G cells with NCF2 knockdown, and in U87 and U251 cells with NCF2 overexpression. **(B)** qRT-PCR analysis confirming changes in *NCF2* and *OPN* mRNA expression in LN229 and T98G cells with NCF2 knockdown, and in U87 and U251 cells with NCF2 overexpression. qPCR was performed in triplicate for each sample. **(C)** Western blot analysis of NCF2, Notch1, and OPN protein expression in LN229 and T98G cells with NCF2 knockdown, with or without Notch1 agonist (N1A) treatment, and in U87 and U251 cells with NCF2 overexpression, with or without Notch1 inhibitor (N1i) treatment. **(D)** qRT-PCR analysis of *NCF2*, *Notch1*, and *OPN* mRNA expression in LN229 and T98G cells with NCF2 knockdown, with or without Notch1 agonist (N1A) treatment, and in U87 and U251 cells with NCF2 overexpression, with or without Notch1 inhibitor (N1i) treatment. qPCR was performed in triplicate for each sample. (“*”represents P<0.05).

Western blot analysis indicated that OPN protein levels were positively correlated with NCF2 expression in GBM cell lines. Specifically, OPN expression was reduced in NCF2 knockdown cells (LN229, T98G) and elevated in *NCF2*-overexpressing cells (U87, U251) (**Figure 5A**). Similarly, qRT-PCR confirmed that *OPN* mRNA levels followed the same trend(LN229si, T98Gsi, U87over, U251over) (NCF2:Change multiplier=0.103,P=0.016; 0.108,P = 0.009; 14,P = 0.000; 12,P = 0.000. OPN: Change multiplier=0.25,P=0.001; 0.25,P = 0.001; 8,P = 0.000; 6.333,P = 0.000.) (**Figure 5B**). To determine the involvement of the *Notch1* signaling pathway, cells were treated with pathway-specific modulators. Administration of a Notch1 agonist (Jagged1) restored Notch1 and OPN expression in *NCF2*-knockdown cells, while treatment with a Notch1 inhibitor (DAPT) significantly suppressed both Notch1 and OPN expression in NCF2-overexpressing cells(LN229si, LN229si+N1A, T98Gsi, T98Gsi+N1A), (NCF2:Change multiplier=0.250,0.277,P=0.644; 0.25,0.27,P = 0.604; Notch1:Change multiplier=0.307,0.783,P=0.000; 0.317,0.863,P = 0.004; OPN: Change multiplier=0.35,0.85,P=0.000; 0.273,0.790,P = 0.001.). (U87over, U87over+N1i, U251over,U251over+N1i) (NCF2:Change multiplier=12.667,11.667,P=0.101; 10.667,10.333,P = 0.685; Notch1:Change multiplier=11.000,2.333,P=0.001; 8.333,1.833,P = 0.000; OPN: Change multiplier=8.000,1.533,P=0.002; 8.000,1.700,P = 0.009.) (**Figures 5C, D**). These findings indicate that NCF2 regulates OPN expression through the activation of the Notch1 signaling pathway.

### 3.6 Blocking OPN reverses NCF2-mediated macrophage M2 polarization

**Figure 6:**
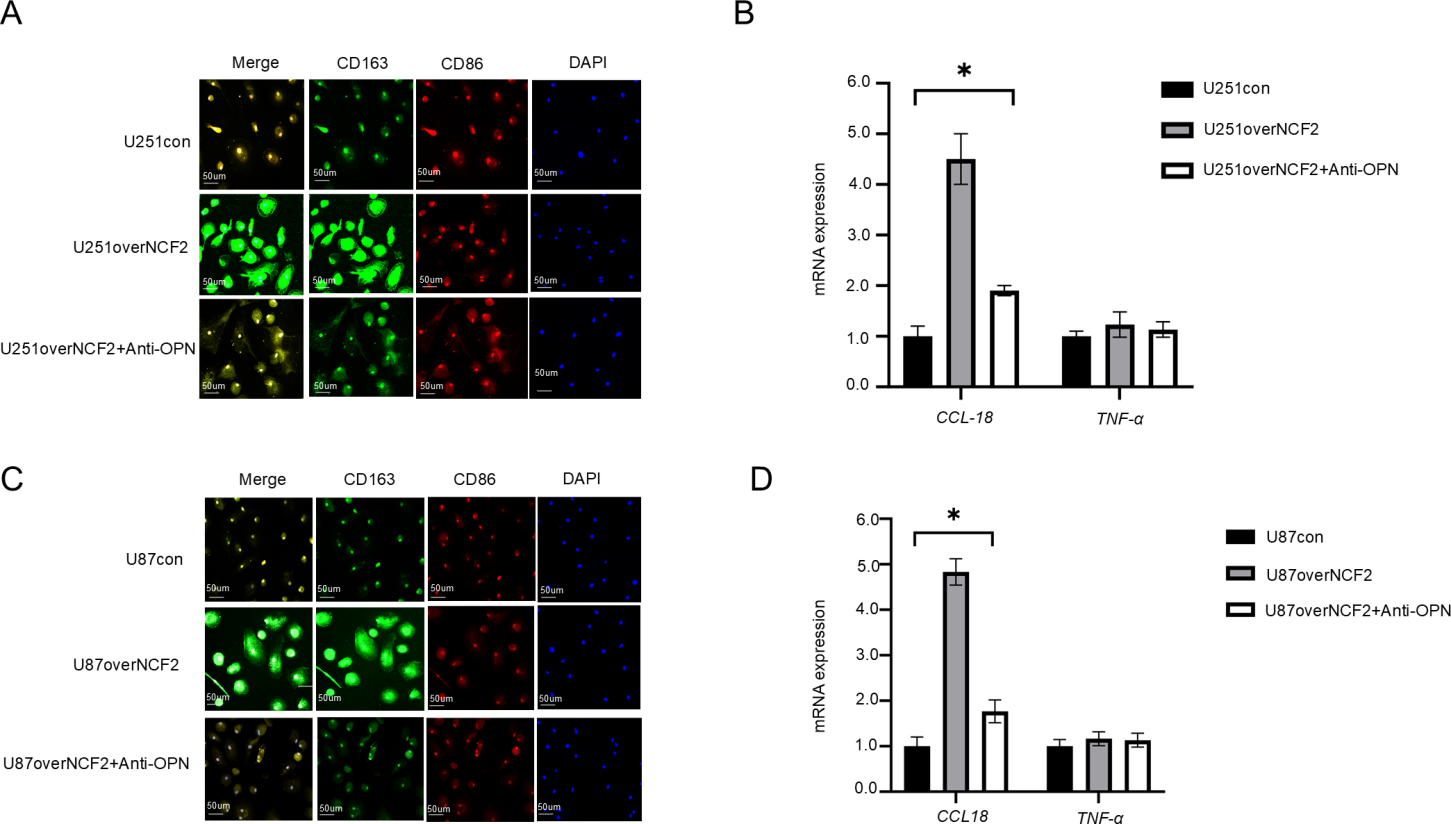
Blocking OPN reverses the effect of NCF2 in promoting macrophage polarization toward the M2phenotype. **(A)** Representative immunofluorescence staining of CD163 (M2 marker) and CD86 (M1 marker) in macrophages differentiated from PBMCs of healthy donors (illustrated in **Supplementary Figure S1**), after 24 h co-culture with conditioned media from U251 control cells, U251 cells overexpressing NCF2, or U251 cells overexpressing NCF2 with OPN neutralizing antibody treatment. **(B)** qRT-PCR analysis of *TNF-α* (M1 marker) and *CCL18* (M2 marker) mRNA expression in macrophages treated as in **(A)**. qPCR was performed in triplicate for each sample. **(C)** Representative immunofluorescence staining of CD163 and CD86 in macrophages after 24 h co-culture with conditioned media from U87 control cells, U87 cells overexpressing NCF2, or U87 cells overexpressing NCF2 with OPN neutralizing antibody treatment. **(D)** qRT-PCR analysis of *TNF-α* and *CCL18* mRNA expression in macrophages treated as in **(C)**. qPCR was performed in triplicate for each sample. (“*”represents P<0.05).

To evaluate the functional role of OPN in NCF2-induced macrophage polarization, PBMC-derived macrophages were cultured with conditioned media from GBM cells. Immunofluorescence staining showed increased CD163 expression in macrophages treated with supernatants from NCF2-overexpressing U251 cells. This effect was significantly attenuated by the addition of a neutralizing anti-OPN antibody. Expression of CD86 remained unaffected across all experimental groups (**Figures 6A, C**). qRT-PCR analysis revealed no significant changes in *TNF-α* mRNA levels among groups. However, *CCL18* expression was elevated in macrophages exposed to conditioned media from *NCF2*-overexpressing and was significantly reduced upon OPN blockade (U251over,U251over+antiOPN,U87over,U87over+antiOPN),(CCL18:Change multiplier=4.5,1.9,P=0.013;4.833,1.767,P=0.000;TNF-α: Change multiplier=1.233,1.133,P=0.598;1.167,1.133,P=0.802.) (**Figures 6B, D**). These findings suggest that OPN functions as a downstream effector of NCF2 in promoting macrophage polarization toward an M2 phenotype.

## 4 Discussion

NADPH oxidase is a major enzymatic source of endogenous ROS in mammalian cells, mediating the reduction of molecular oxygen to form ROS (10). As critical byproducts of aerobic metabolism, ROS play multifaceted roles in tumor biology, including the promotion of oncogenic transformation, angiogenesis, invasion, and metastasis. NCF2, a cytoplasmic regulatory subunit of the NADPH oxidase complex, has been identified as a key regulator of ROS production and has been implicated in the pathogenesis of various malignancies (11–13). The expression and activation of NCF2 are potentially regulated by upstream factors including the transcription factors C/EBPϵ for its gene expression, and the small GTPase Rac and membrane translocation for its functional activation (14–16). Previous studies have reported that elevated NCF2 expression is correlated with poor prognosis in renal cell carcinoma (17), modulation of chemokine signaling and immune infiltration in prostate cancer (18), and diagnostic as well as prognostic utility in cervical squamous cell carcinoma (19). In hepatocellular carcinoma, NCF2 overexpression is linked to altered immune infiltration and worse survival (3), while in melanoma, mutations in NCF2 affect responsiveness to immunotherapy (20). Bioinformatics analyses have further indicated that NCF2 may contribute to radioresistance and reduced survival in GBM (21).

Consistent with these findings, the present study demonstrated that NCF2 expression was significantly elevated in GBM tissues compared to normal brain tissues, as confirmed by IHC analysis, western blotting, and qRT-PCR. GBM cell lines (U251, U87, LN229, and T98G) also displayed markedly higher NCF2 expression compared to normal human astrocytes, supporting the notion that NCF2 upregulation occurs at both tissue and cellular levels. While our study’s findings based on 18 GBM specimens provide valuable mechanistic insights, the moderate sample size may limit statistical power and generalizability, necessitating future validation in larger cohorts.

Although the association between NCF2 and tumor progression has been observed across several cancer types, mechanistic insights into its functional role, particularly in GBM, remain limited. TAMs constitute a major immune cell population within the glioma microenvironment and are involved in supporting tumor progression (22). TAMs exhibit remarkable plasticity, polarizing into either classically activated (M1) macrophages or alternatively activated (M2) macrophages. M1 macrophages exert pro-inflammatory and anti-neoplastic effects and are characterized by elevated expression of TNF-α, NOS2, IL1B, IL6, CXCL9/10, and the surface marker CD86. Conversely, M2 macrophages contribute to immunosuppression and tumor promotion, with increased expression of CCL18, MRC1 (CD206), MSR1 (CD204), IL10, TGFB1, and surface marker CD163 (23, 24).

In the context of glioma, TAMs are predominantly polarized toward the M2 phenotype, facilitating an immunosuppressive microenvironment that supports tumor growth and evasion of immune surveillance (7, 8). In the current study, a significant positive correlation was observed between NCF2 expression and infiltration of CD163^+^ TAMs in GBM tissue samples. Furthermore, functional co-culture assays confirmed that conditioned media derived from NCF2-overexpressing GBM cells promoted CD163 and CCL18 expression in monocyte-derived macrophages, while NCF2 knockdown reduced these markers. Notably, CD86 and TNF-α expression remained unchanged, suggesting that NCF2 selectively promotes macrophage polarization toward the M2 phenotype. These findings highlight provide mechanistic evidence that NCF2 contributes to the immunosuppressive remodeling of the GBM microenvironment by enhancing M2 macrophage polarization.

At the mechanistic level, this study identified OPN, encoded by *SPP1*, as a downstream effector of NCF2. OPN is a multifunctional, secreted glycoprotein produced by various cell types and is markedly upregulated in multiple malignancies (25). It has been implicated in several aspects of tumor biology, including the promotion of tumor progression and the facilitation of macrophage polarization toward the M2 phenotype (26). Prior studies have demonstrated that activation of the Notch1 signaling pathway enhances OPN expression and have identified *SPP1* as a direct transcriptional target of Notch1 (27, 28). The Notch signaling pathway, composed of four receptors (NOTCH1–4) and five canonical ligands (Jagged1–2, DLL1, DLL3, DLL4), is evolutionarily conserved and functionally significant in gliomagenesis. In particular, it plays a critical role in maintaining glioma cell stemness, proliferation, and self-renewal capacity (29).

In the present study, a strong positive correlation between NCF2 and OPN expression was observed in GBM tissue samples. Furthermore, modulation of Notch1 activity altered OPN expression in GBM cells with manipulated NCF2 levels. Activation of Notch1 restored OPN expression in NCF2-knockdown cells, whereas inhibition of Notch1 suppressed OPN expression in NCF2-overexpressing cells. These findings suggest that NCF2 regulates OPN expression via the Notch1 signaling pathway. Importantly, functional assays demonstrated that blocking OPN abrogated the ability of NCF2-overexpressing GBM cells to induce M2 polarization in macrophages. This was evidenced by decreased expression of CD163 and CCL18, without affecting M1-associated markers. These findings support a mechanistic model in which NCF2 promotes immunosuppressive macrophage polarization via the activation of the Notch1/OPN axis in the GBM microenvironment. Targeting the NCF2/OPN axis in glioblastoma represents a promising combinatorial strategy that may enhance the efficacy of emerging immunotherapies such as immune checkpoint inhibitors and adoptive cell therapies by reprogramming the immunosuppressive tumor microenvironment, thereby offering a synergistic approach to overcoming therapeutic resistance.

## 5 Conclusion

This study provides novel evidence that NCF2 is not only overexpressed in GBM but also functionally contributes to the immunosuppressive tumor microenvironment by promoting M2 macrophage polarization. At the mechanistic level, NCF2 regulates OPN expression via activation of the Notch1 signaling pathway. The identified NCF2–Notch1–OPN axis plays a critical role in driving M2 macrophage differentiation within the GBM microenvironment. These findings enhance current understanding of the oncogenic functions of NCF2 in GBM and underscore its potential as a biomarker for disease progression. More importantly, therapeutic targeting of the NCF2–Notch1–OPN signaling cascade may offer a promising therapeutic strategy for modulating TAM polarization and attenuating glioma progression. Further *in vivo* and translational studies are warranted to validate these observations and to evaluate the clinical feasibility of interventions targeting this pathway.

## Data Availability

The original contributions presented in the study are included in the article/**Supplementary Material**. Further inquiries can be directed to the corresponding author.

## Supplementary material

Supplementary Figure 1 Isolation of peripheral blood mononuclear cells (PBMCs) and M-CSF–induced differentiation into macrophages. (A) Representative bright-field image of PBMCs freshly isolated from healthy donor peripheral blood by density-gradient centrifugation (Ficoll).(B) Monocyte-derived macrophages after 7 days of culture with M-CSF (50 ng/mL), showing adherent, enlarged, and spread morphology.(C) Immunofluorescence confirmation of macrophage identity by CD68 staining (CD68, green) with nuclear counterstain (DAPI, blue).

Supplementary Figure 2 Acquisition and Analysis of Bioinformatics Data: Correlation between NCF2 and OPN. GEPIA is an interactive analysis platform based on gene expression level values. On this platform, a significant correlation (P = 4.4e-16,R=0.58) was found between the mRNA expression levels of NCF2 and OPN.

Supplementary Figure 3 Acquisition and Analysis of Bioinformatics Data: Correlation between NCF2 and CD63. GEPIA is an interactive analysis platform based on gene expression level values. On this platform, a significant correlation (P = 0.00,R = 0.72) was found between the mRNA expression levels of NCF2 and CD163.
