# Supplementary figures and images for: NCF2 facilitates M2 macrophage polarization in glioblastoma through activation of the notch1–osteopontin axis
Source: Front Immunol. 2026 Feb 12;17:1743950. doi: 10.3389/fimmu.2026.1743950 (PMC12936047; doi:10.3389/fimmu.2026.1743950)

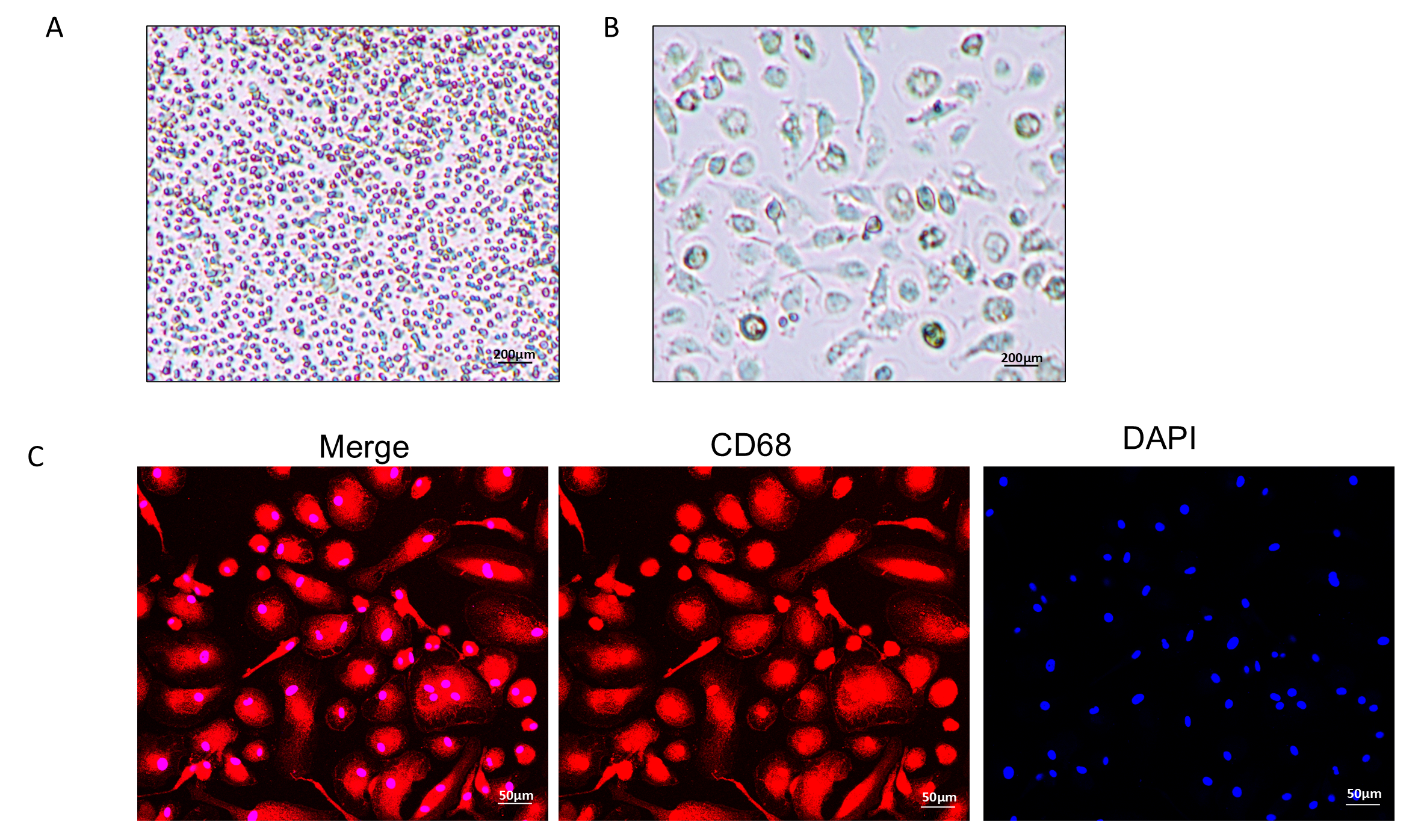

Supplement: Supplementary Figure 1 — Isolation of peripheral blood mononuclear cells (PBMCs) and M-CSF–induced differentiation into macrophages. (A) Representative bright-field image of PBMCs freshly isolated from healthy donor peripheral blood by density-gradient centrifugation (Ficoll).(B) Monocyte-derived macrophages after 7 days of culture with M-CSF (50 ng/mL), showing adherent, enlarged, and spread morphology.(C) Immunofluorescence confirmation of macrophage identity by CD68 staining (CD68, green) with nuclear counterstain (DAPI, blue). [file Image1.tif]

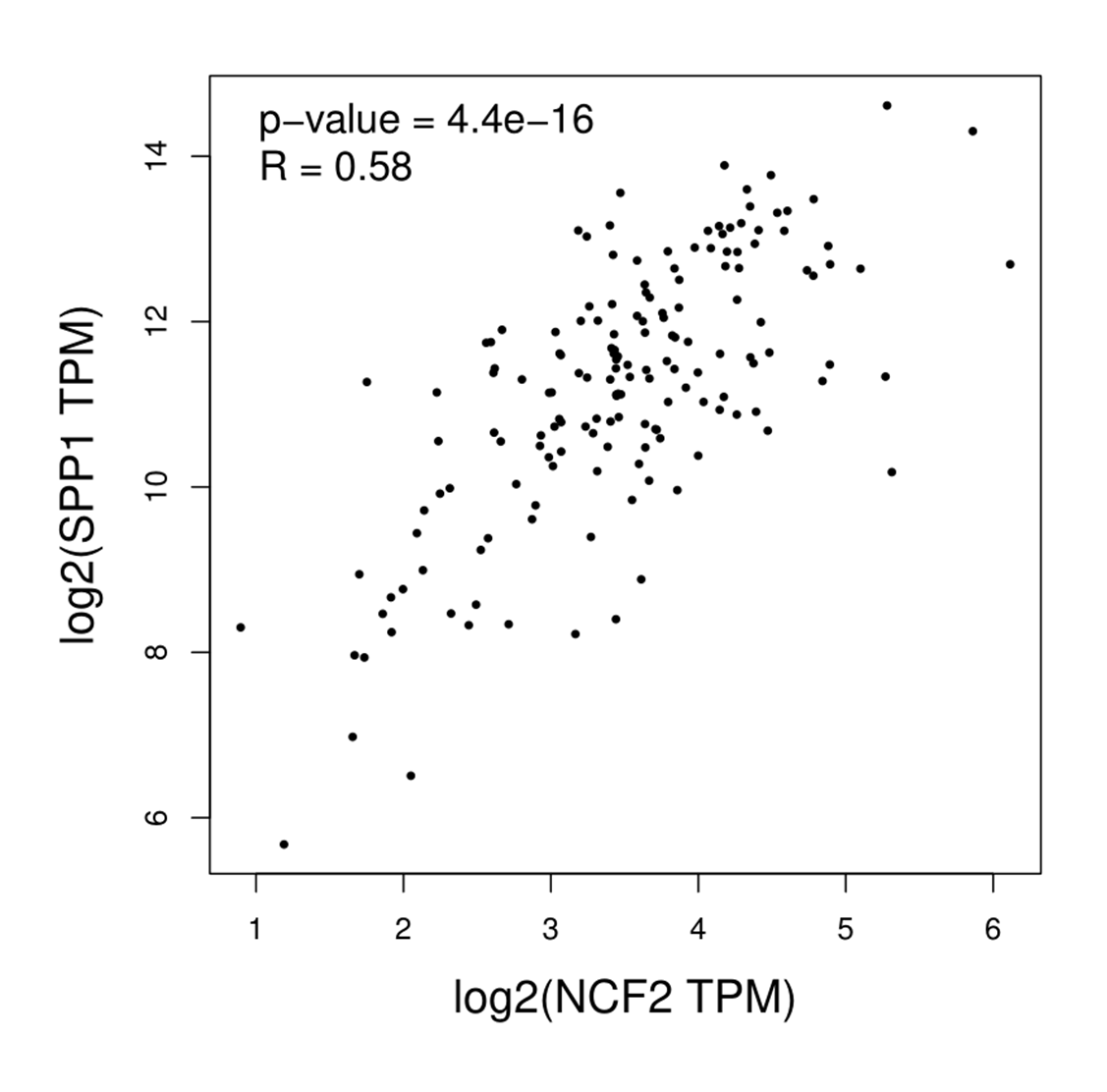

Supplement: Supplementary Figure 2 — Acquisition and Analysis of Bioinformatics Data: Correlation between NCF2 and OPN. GEPIA is an interactive analysis platform based on gene expression level values. On this platform, a significant correlation (P = 4.4e-16,R=0.58) was found between the mRNA expression levels of NCF2 and OPN. [file Image2.tif]

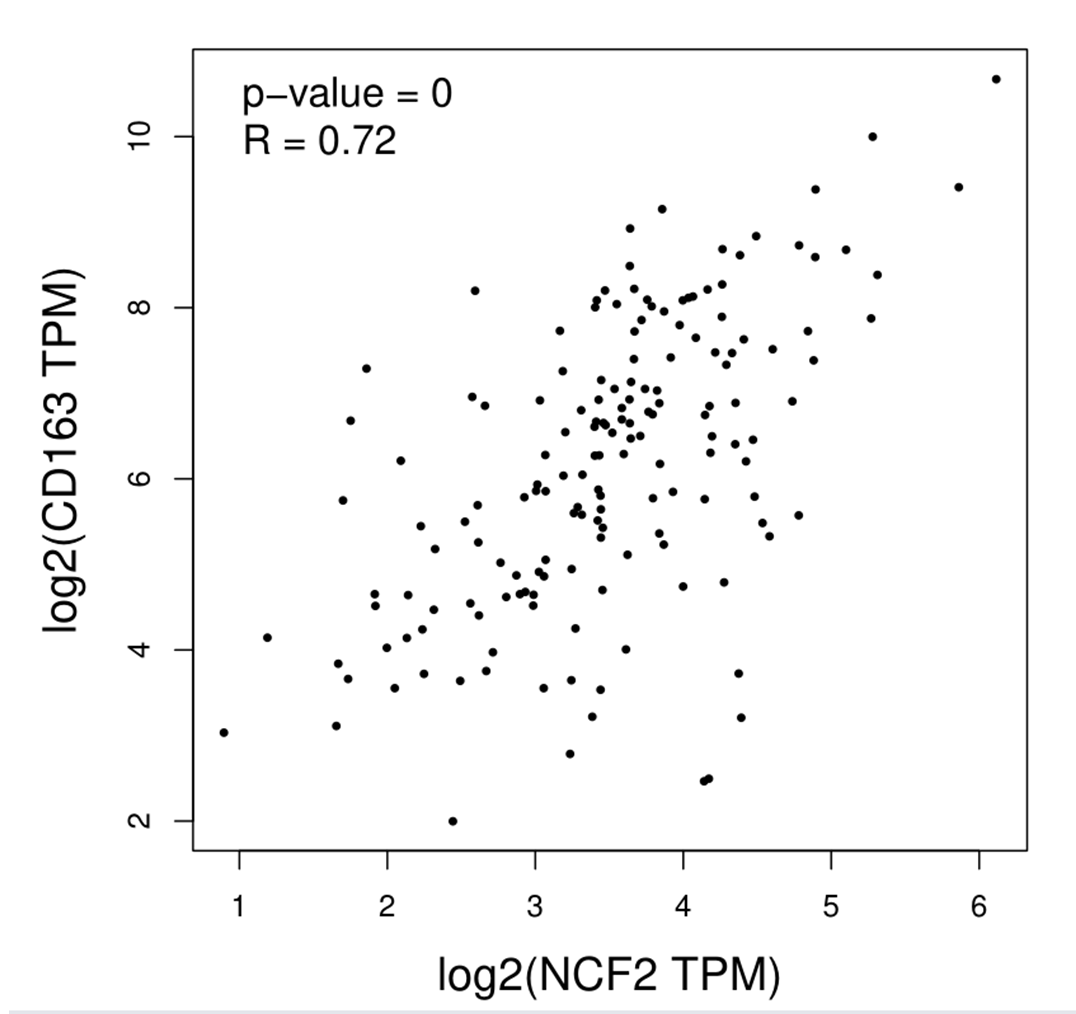

Supplement: Supplementary Figure 3 — Acquisition and Analysis of Bioinformatics Data: Correlation between NCF2 and CD63. GEPIA is an interactive analysis platform based on gene expression level values. On this platform, a significant correlation (P = 0.00,R = 0.72) was found between the mRNA expression levels of NCF2 and CD163. [file Image3.tif]
